# Improving Comparative Effectiveness Research of Complex Health Interventions: Standards from the Patient-Centered Outcomes Research Institute (PCORI)

**DOI:** 10.1007/s11606-020-06093-6

**Published:** 2020-10-26

**Authors:** Laura C. Esmail, Rebecca Barasky, Brian S. Mittman, David H. Hickam

**Affiliations:** 1 Clinical Effectiveness and Decision Science Program, Patient-Centered Outcomes Research Institute (PCORI), Washington, DC USA; 2 Washington, DC USA; 3 Health Services Research & Implementation Science, Department of Research & Evaluation, Kaiser Permanente Southern California, Pasadena, CA USA

**Keywords:** complex interventions, complex health interventions, comparative effectiveness research, patient-centered outcomes research

## Abstract

**Introduction:**

Complex health interventions (CHIs) are increasingly studied in comparative effectiveness research (CER), and there is a need for improvements in CHI research practices. The Patient-Centered Outcomes Research Institute (PCORI) Methodology Committee (MC) launched an effort in 2016 to develop formal guidance on this topic.

**Objective:**

To develop a set of minimal standards for scientifically valid, transparent, and reproducible CER studies of CHIs. The standards are intended to apply to research examining a broad range of healthcare interventions including delivery system, behavior change, and other non-pharmacological interventions.

**Methods:**

We conducted a literature review, reviewed existing methods guidance, and developed standards through an iterative process involving the MC, two panels of external research methods experts, and a 60-day public comment period. The final standards were approved by the PCORI MC and adopted by the PCORI Board of Governors on April 30, 2018.

**Results:**

The final standards include the following: (1) fully describe the intervention and comparator and define their core functions, (2) specify the hypothesized causal pathways and their theoretical basis, (3) specify how adaptations to the form of the intervention and comparator will be allowed and recorded, (4) plan and describe a process evaluation, and (5) select patient outcomes informed by the causal pathway.

**Discussion:**

The new standards offer three major contributions to research: (1) they provide a simple framework to help investigators address the major methodological features of a CHI study, (2) they emphasize the importance of the causal model and the need to understand *how* a CHI achieves its effects rather than simply measuring these effects, and (3) they require description of a CHI using the concepts of core functions and forms. While these standards apply formally to PCORI-funded CER studies, they have broad applicability.

**Electronic supplementary material:**

The online version of this article (10.1007/s11606-020-06093-6) contains supplementary material, which is available to authorized users.

## INTRODUCTION

Complex health interventions (CHIs) are central to efforts to improve healthcare and patient outcomes. CHIs are generally defined as multicomponent, adaptable interventions that act independently or interdependently to change care processes and outcomes and typically require specific involvement and behaviors by patients, caregivers, and health professionals.^1, 2^ Examples include delivery system interventions, behavioral and psychological interventions, and other non-pharmacological treatments.

The Patient-Centered Outcomes Research Institute (PCORI) has funded a large research portfolio examining the comparative effectiveness of CHIs. PCORI was established to produce and promote high-integrity, evidence-based information from research guided by patients, caregivers, and the broader healthcare community. PCORI provides scientific guidance for research through a set of Methodology Standards.^3, 4^ The Methodology Standards are intended to ensure that the research funded by PCORI is of high quality and rigorous. The standards are used by merit reviewers in the assessment of funding applications, by PCORI staff in monitoring funded studies, and by external peer reviewers to evaluate final research results.

The intent of the PCORI Methodology Standards is to define the minimal requirements for valid research (rather than offering “aspirational” guidance). Because of this emphasis on minimal guidance, the standards are highly focused and often less expansive than guidance provided by other sources. The standards do not specify which specific research questions or study designs (such as a randomized controlled trial or an observational design) should be pursued. Instead, the standards recommend approaches for ensuring the integrity and validity of the study after a research topic and high-level design have been chosen. The standards comprise a broad-based set of recommendations grouped in inter-related categories. For example, one category provides guidance on ensuring the integrity of the collected data, while another addresses appropriate analyses of those data for assessing causal inference hypotheses. The standards have been continuously expanded and updated since they were first released in 2012.

Given the frequency with which CHIs are studied in the field of patient-centered outcomes research (PCOR) and the acknowledged need for improvements in CHI research practices,^5, 6^ in 2016, the PCORI Methodology Committee launched an effort to develop formal guidance on this topic. The new standards were developed and reviewed over a period of 2 years. While these new standards apply to PCORI-funded research, they have broad applicability to studies of CHIs supported by other funding agencies across numerous categories of health-related research.

## OBJECTIVE

We aimed to develop a set of methodological standards to ensure the scientific validity, transparency, and reproducibility of studies of complex health interventions in PCOR. This article describes the process of developing these standards, the major themes, and the rationale supporting them.

## METHODS

**Figure 1:**
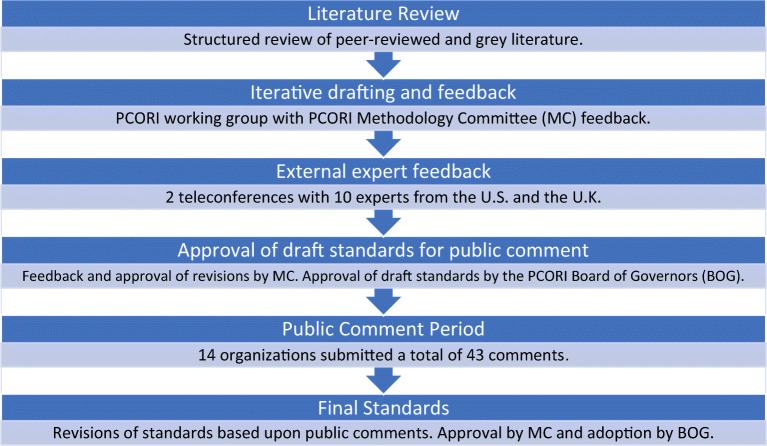
Process of development of the standards**.**

Development of methodology standards is a core role of the PCORI Methodology Committee (MC), a standing committee of experts in clinical, behavioral, and health services research.^7^ PCORI staff and MC members developed the standards through a stepwise process depicted in Fig. 1.

To initiate our work, we formed an internal PCORI staff working group led by a MC member who is an expert in the field of complex health interventions (BSM). We performed an initial literature review to identify seminal articles, including guidelines, frameworks, and reviews that address the design and methods for studies evaluating complex health interventions. Our procedure for the literature review is outlined in Appendix 1. Subsequently, the working group followed an iterative process of drafting and discussion with a designated subgroup of the MC, which included methodologists with backgrounds in implementation science, health services research, and surgical clinical trials. The MC subgroup provided feedback, revisions, and guidance throughout the 18-month development process. The entire MC regularly reviewed drafts of the developing standards during in-person and teleconference meetings. After obtaining MC approval of the second complete draft, we held two teleconferences with separate groups of national and international experts to discuss and provide feedback on the standards (see Acknowledgments). Each teleconference had a duration of three hours. The expert reviews guided additional revisions followed by the release of draft standards for a 90-day public comment period.

PCORI staff reviewed and addressed each comment by either incorporating further revisions or explaining why revisions were not introduced.^8^ The MC reviewed the responses and provided feedback to PCORI staff prior to finalizing revisions. The MC voted unanimously to approve the final standards in March 2018, and the PCORI Board of Governors voted to adopt the final standards for public release on April 30, 2018.

## RESULTS

**Figure 2:**
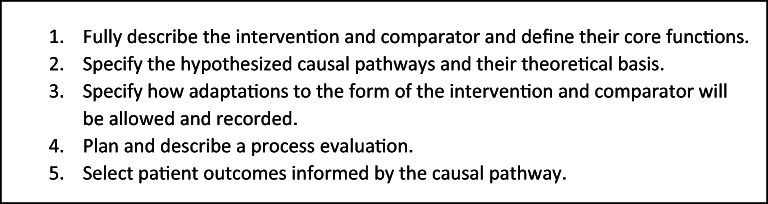
Final PCORI methodology standards for studies of complex health interventions**.**

The activities to develop the standards led to three successive drafts. The initial draft standards were based on the literature review of existing guidance on CHIs, which covers a range of aspects including intervention development, intervention description, and planning for and/or reporting on types of CHI evaluations (e.g., process evaluation, effectiveness studies, cost-effectiveness studies, implementation studies, and systematic reviews). While we pulled from these diverse sources, our standards retained focus on the evaluation of comparative effectiveness studies of CHIs, in line with PCORI’s mission. The initial draft standards addressed six themes: (1) specification of a conceptual framework, (2) description of the intervention, (3) process evaluation, (4) selection of outcome measures, (5) analytical approaches, and (6) reporting. Some issues featured in existing guidance (such as sample size considerations and choice of study design)^1^ were not included, so as to be consistent with the overall focus of the PCORI Methodology Standards. Input from the MC and external experts then led to introduction of new themes, consolidation, and re-organizing the standards. Public posting of a preliminary version of the standards yielded a total of 43 comments on the draft standards. The final set of standards (Fig. 2) was posted and explained on the PCORI website following their authorization by the PCORI Board of Governors. Appendix 2 provides the expanded wording for the standards that was disseminated to the public.^9^

The final group of five standards outlines the key elements that researchers should consider, describe, and justify a priori when designing studies to evaluate complex interventions. The main differences in these standards compared with existing guidance include the requirements to describe the CHI in terms of core functions and forms, explicitly articulate/visualize the CHI’s hypothesized causal pathways, and pre-specify expected adaptations. The sections below describe each standard and outline the supporting rationale.

### Fully Describe the Intervention and Comparator and Define Their Core Functions

The first standard aims to address the recurring problem of inadequate description of interventions by requiring investigators to describe fully the intervention and comparator(s) that they propose to study. The problem of inadequate description may involve insufficient detail, use of inconsistent terminology, or a lack of specification of the intervention’s intended purpose(s).^10, 11^

This standard extends existing guidance by requiring description of the intervention in terms of core functions and forms. The term *forms* refers to the details of the intervention components and activities, which reflects existing guidance.^1, 11–13^ The term *core functions* adapts a construct introduced by Hawe et al. that describes a complex health intervention primarily in terms of the CHI’s core purpose(s) rather than its components or activities.^14–16^ A core function is the fundamental purpose or desired effect (on patients, health professionals, or staff behaviors) of a set of CHI activities. For example, in a diabetes self-management program, a core function could be ensuring communication between patients and providers about home measurements of blood glucose levels. Likewise, a core function of cognitive behavioral therapy (CBT) is training patients in problem-solving and coping skills. For this second example, the form comprises the activities carried out to achieve the training goal, including the content, number and length of sessions, qualifications of the provider who delivers CBT, and how it is delivered (e.g., group, individual, online, in-person). In the context of an effectiveness study, it is critical to ensure fidelity to function rather than to form: the form(s) selected for each core function may vary based on contextual factors influencing the feasibility or appropriateness of selected forms.^14, 17^ In PCOR and CER, fidelity to core functions is critical to ensure that the same intervention is being implemented and studied across all sites and settings, whereas selection of forms (specific details of the activities for carrying out each function) may vary. As the field rapidly advances, several examples have emerged that operationalize these concepts.^16, 18–22^

The core function-form approach contrasts with conventional approaches to defining complex health interventions in terms of core components, which assumes that the forms of an intervention—details of content or delivery—are critical to achieving its effects. The conventional approach fails to consider the importance of the causal pathway and mechanisms of effect and the likelihood that alternative means are available to achieve the desired outcome. Prioritizing core components and introducing manualized interventions with highly specified (and often idiosyncratic) activities lead to several problems including (1) precluding beneficial tailoring of CHIs in settings where resource constraints and other contextual factors require modification, (2) lack of explicit attention to a CHI’s fundamental mechanisms of effect and how various modifications strengthen or compromise a CHI’s effectiveness, (3) errors in measuring fidelity (to form rather than function), and (4) the inability to replicate or scale up study results.^15, 18^

### Specify the Hypothesized Causal Pathways and Their Theoretical Basis

We developed the second standard to emphasize the importance of specifying explicit hypothesized causal pathways in the planning phase of the study.^1, 13, 23^ A causal pathway describes the sequence of events and mechanisms of effect that produce changes in care processes and, ultimately, patient outcomes. The causal pathways vary based on the health issue under study and the intended effects of the CHI. Causal pathways should be based on relevant theory and capture the core functions of the intervention under study. Terms such as “conceptual framework,” “logic model,” and “theory of change” often fail to convey the level of detail or explicit commitment to identified (hypothetical or empirically supported) effects upon patients. This second standard requires explicit description of the causal pathway as well as sufficient information to assess its justification. Researchers should explain how the causal pathway is based on previously described theories or provide empirical evidence to support the CHI’s hypothesized effects. The description also should describe how the forms or activities carrying out the core functions are expected to interact and achieve their effects.

The requirement for specification of causal pathways aligns with existing themes of the PCORI Methodology Standards. The previously developed standards on causal inference require researchers to specify the causal model underlying the research question.^9^ The causal pathway should depict the intervention’s functions and how each function explicitly interacts in a chain of events leading to the predicted change in patient outcomes.^24^ It also should include key contextual factors that may facilitate or reduce a CHI’s effectiveness. These include both internal and external factors.^25^ Internal factors can include organizational policies and procedures, availability of resources (including qualified staff), and organizational culture and norms.^26^ External factors can include regulatory influences, regional and national professional influences, and local social/cultural influences. Each of these factors can directly influence the outcomes targeted by a CHI (e.g., community regulations or social norms discouraging smoking that augment health system-based smoking cessation initiatives) and can exert indirect influences by affecting the specific form of the CHI or the extent of its implementation or delivery (e.g., scope-of-practice regulations governing nurse prescribing privileges).

The hypothesized causal pathway must be linked to its theoretical basis and supported with empirical data when possible. Making the causal assumptions explicit at the study planning stage provides clarity about issues such as the selection of appropriate comparators, patient outcomes, as well as the identification of key covariates, mediators, and moderators to measure.^1^

### Specify How Adaptations to the Form of the Intervention and Comparator Will Be Allowed and Recorded

The third standard requires investigators to pre-specify permitted or expected adaptations to the CHI that may be required to facilitate implementation and enhance effectiveness of the complex health intervention in a given setting. These expected adaptations must preserve the integrity (core functions) and enhance effectiveness of the intervention. Contextual factors—anything external to the intervention that may facilitate or impede its implementation—often limit the feasibility of a standardized form of a CHI.^13, 23^ Issues such as time, resources, training, organizational context, language, and culture may necessitate that CHIs be tailored to accommodate the needs and circumstances of the local setting and population.^27^ Too often, study sites will introduce adaptations in an ad hoc manner where the potential impacts of the changes on the effectiveness of the intervention are not fully considered or well understood and where they reduce fidelity to the underlying core functions. This third standard requires investigators to consider and plan anticipated adaptations to the form of the intervention, guided by explicitly identified core functions, and to formalize this in a protocol.^1, 12, 28^

Specifying adaptations at the study design stage requires an understanding of the core functions of the intervention and the variations in form that will maintain fidelity to these core functions.^29^ Ideally, adaptations will be supported with empirical evidence (from prior research on the same or similar interventions or pilot study) but at a minimum, they need to have a clear rationale and be supported by theory. Permissible adaptations should be (1) responsive to local conditions to facilitate implementation and enhance effectiveness of the intervention when implemented in each setting and (2) guided by the core function. Fidelity to an intervention’s core functions is critical to assure the integrity of the fundamental purpose and intended effects of the intervention.

Unintended adaptations—adaptations to CHIs implemented by researchers or healthcare staff that were unplanned and improvised—will likely occur. The study protocol should include procedures to track what changes are made, who made them, the frequency with which they are made, the reasons for which they were made, and whether they are consistent with the intervention’s pre-identified core functions.^28^ Identifying expected and potential adaptations at the study planning stage permits the researcher to consider the appropriate data collection points and approaches to manage, document/measure, and report both planned and unplanned adaptations.

### Plan and Describe a Process Evaluation

Some complex health interventions may have relatively small impacts on the outcomes experienced by patients. It is thereby important to learn whether the measured outcomes are due to relatively low impact of the intended core functions or rather that the intended core functions were not actually achieved. Distinguishing between these two alternative explanations is important when interpreting studies evaluating CHIs.^13^ The fourth standard addresses this need by advising investigators to plan and conduct a process evaluation to determine whether and how an intervention achieved its intended effects. Process evaluations provide key information about the expected impact and mechanisms of effect of a complex health intervention. They can inform future researchers’ and decision-makers’ efforts to reproduce and tailor the intervention and apply the results to their own settings and patient populations. Process evaluations can also help elucidate how an intervention’s effectiveness may be altered by the setting and contextual factors and therefore help explain differences between expected and observed outcomes.^23, 30^

An essential feature of a process evaluation is to identify measurable intermediate outcomes that are related to achievement of the intervention’s stated core functions. The causal pathway (which provides the rationale for the core functions) should drive the identification of process questions and data collection.^23^ A process evaluation needs to be carefully planned and scoped, to target data collection narrowly and to minimize participant burden.

Interpretation of process findings can be enhanced by further information about the ways in which the functions were carried out. This involves how the CHI is implemented and its alignment with what was originally planned in the study’s protocol.^12^ The fourth standard requires investigators to measure and report the fidelity of delivery, adaptations (planned and unplanned), and dose or quantity of the CHI forms or activities.^11, 12, 23, 28^ Measuring and reporting how a participant responds to and interacts with a CHI (i.e., mechanisms of effect) may identify causal mechanisms that had not previously been recognized. Furthermore, failure to achieve an intervention’s core functions can sometimes be plausibly attributed to contextual factors, which should be considered when determining what process-related data to collect.^25, 31^

The standards are agnostic regarding which methods or analytic techniques can be used, as it depends upon the specific research question at hand. Both quantitative and qualitative evaluations can be chosen, with the goal being a strong overall evaluation that provides insights into mechanisms of effect and the challenges encountered in carrying out the CHI.^32, 33^ Quantitative methods generally are well suited for measuring fidelity, dose, and reach and testing hypothesized mechanisms of effect (mediators and mediation processes), while qualitative approaches are useful in determining how participants experience the intervention as well as providing descriptive information on participants’ views of causal mechanisms.^21, 23, 34–36^

### Select Patient Outcomes Informed by the Causal Pathway

The fifth standard requires investigators to measure outcomes relevant and important to the population of interest. Although process evaluations are essential for understanding the operation and effects of a CHI, they are often limited in the ability to provide information about an intervention’s ultimate impact on patients. The patient outcomes are derived from the causal model and should be measured at a time point appropriate for understanding how an intervention affects patients.^1^

## DISCUSSION

Development of the PCORI Methodology Standards on CHIs was based on the approach PCORI has used for several years to provide broad guidance for researchers and users of research results. These standards are derived from recommendations previously published by other groups but provide a unique synthesis of the essential issues. Considerable effort was devoted to ensuring that the PCORI guidance correctly synthesizes and aligns with the latest methodological developments while reconciling any contradictions in published recommendations. The PCORI standards are also intended to be “minimal.” They define the essential components of valid research on CHIs but are not intended to be a comprehensive summary nor a detailed guide to all possible approaches to such research. The new standards offer three major contributions to the field: (1) they provide a simple framework to help investigators think through the major methodological aspects of their CHI study, (2) they emphasize the importance of the causal model and the need to understand *how* a CHI achieves its effects rather than simply measuring these effects, and (3) and they formalize the description of CHIs in terms of core functions and forms. While these standards apply formally to PCORI-funded CER studies, they have broad applicability.

These standards focus on basic principles that are the foundation of good clinical, behavioral, and health services research. The standards state that researchers should describe and justify the study’s approach at the initial phase of research conception and design through the final phases of implementation and reporting. We pursued a balance between providing enough guidance to ensure basic issues are addressed, without requiring researchers to follow a specific method or approach. Ample guidance exists elsewhere regarding how to operationalize the concepts outlined in these standards. Our focus on key principles contributes to the field by providing a straightforward approach to think through the design of complex health intervention studies.

Our work led to the conclusion that the causal model is the foundation of any complex health intervention study—as is the case in most rigorous scientific research. It is not that the causal model is more important in research on complex health interventions. The issue is that the causal model has often been poorly described, if described at all, and too often not appropriately used to guide study design, conduct, and interpretation. By definition, CHIs involve multiple interacting activities as well as interactions among patients, health professionals, and other agents in the healthcare system. A CHI is variable in its implementation and extremely sensitive to context. These characteristics make defining the causal pathways difficult but especially important. Failure to do so can lead to poorly informed study decisions that do not reflect the fundamental underlying assumptions of how the intervention is hypothesized to work.

These new standards are the first to integrate the concepts of core function and form into formal guidance for the study of complex health interventions. Furthermore, by emphasizing fidelity to core function rather than to form, these standards aim to link the underlying purposes or goals of an intervention (rather than its content or activities) to their role in the causal pathway. This may lead to more consistency in how these interventions are understood, described, implemented, and categorized. Another major contribution of the CHI standards is the requirement to identify planned adaptations a priori. While it is unrealistic to expect that all adaptations can be identified in advance, the purpose of this guidance is that the approaches to adaptation can generally be pre-defined and incorporated into the overall plans for the study.

The inclusion of a process evaluation as a core part of a CER study suggests a shift from documenting and comparing to fully understanding and explaining outcomes in CER when applied to complex health interventions. Often, the direct comparison of two or more active CHIs will yield small differences in average effect size. In line with PCORI’s interest in understanding the heterogeneity of treatment effects, a well-designed process evaluation integrated into a comparative effectiveness study of CHIs begins to answer the critical questions of what worked for whom and why.

Our work has limitations. First, we used a systematic and iterative process to develop the CHI standards, but we did not follow a formal deliberative process (e.g., Delphi) to reach consensus on the standards. We followed PCORI’s process for Methodology Standards development, which integrates multiple opportunities for input and feedback on the work, including involvement of the Methodology Committee, the PCORI Board of Governors, external experts, and public comments. The PCORI Methodology Standards are an evolving form of guidance, to accommodate methodological advances, as well as new and updated guidance.^37^ Second, because the standards are not highly prescriptive, they rely on the expertise of investigators to apply them within the context of specific studies. While researchers are encouraged to seek and consult existing guidance, research teams should incorporate relevant expertise from the earliest planning stage of the study. It is the process of thinking about each standard as it relates to the evaluation at hand that can help mitigate common pitfalls and oversights. PCORI has initiated activities to disseminate these standards and provide educational resources to support their implementation. It will be important to integrate these efforts with initiatives undertaken by the broader community of CHI researchers.

## Electronic supplementary material

ESM 1 (DOCX 28 kb)

ESM 2 (DOCX 19 kb)

## References

[1] Craig P, Dieppe P, Macintyre S, Michie S, Nazareth I, Petticrew M (2008). Developing and evaluating complex interventions: the new Medical Research Council guidance. BMJ (Clin Res Ed).

[2] Guise JM, Chang C, Butler M, Viswanathan M, Tugwell P (2017). AHRQ series on complex intervention systematic reviews—paper 1: an introduction to a series of articles that provide guidance and tools for reviews of complex interventions. J Clin Epidemiol.

[3] The PCORI Methodology Committee (2019). The PCORI Methodology Report.

[4] The Methodology Committee of the Patient-Centered Outcomes Research Institute (PCORI) (2012). Methodological standards and patient-centeredness in comparative effectiveness research: the PCORI perspective. JAMA..

[5] Moore G, Audrey S, Barker M, Bond L, Bonell C, Cooper C (2014). Process evaluation in complex public health intervention studies: the need for guidance. J Epidemiol Community Health.

[6] **Richards DA, Hallberg IR.** Complex Interventions in Health: an Overview of Research Methods: Routledge; 2015.

[7] PCORI. Methodology Committee. 2019. https://www.pcori.org/about-us/governance/methodology-committee. Accessed 1 Sept 2019.

[8] The PCORI Methodology Committee. Appendix B: Response to public comment. The PCORI Methodology Report 2019. p. 73-82.

[9] PCORI. PCORI Methodology Standards. 2019. https://www.pcori.org/research-results/about-our-research/research-methodology/pcori-methodology-standards. Accessed 1 Sept 2019.

[10] Michie S, Fixsen D, Grimshaw JM, Eccles MP (2009). Specifying and reporting complex behaviour change interventions: the need for a scientific method. Implement Sci IS.

[11] Hoffmann TC, Glasziou PP, Boutron I, Milne R, Perera R, Moher D (2014). Better reporting of interventions: Template for Intervention Description and Replication (TIDieR) checklist and guide. BMJ (Clin Res Ed).

[12] Mohler R, Kopke S, Meyer G (2015). Criteria for Reporting the Development and Evaluation of Complex Interventions in healthcare: revised guideline (CReDECI 2). Trials..

[13] Guise JM, Butler M, Chang C, Viswanathan M, Pigott T, Tugwell P (2017). AHRQ series on complex intervention systematic reviews—paper 7: PRISMA-CI elaboration and explanation. J Clin Epidemiol.

[14] Hawe P, Shiell A, Riley T (2004). Complex interventions: how “out of control” can a randomised controlled trial be?. BMJ (Clin Res Ed).

[15] Hawe P (2015). Lessons from complex interventions to improve health. Annu Rev Public Health.

[16] Perez Jolles M, Lengnick-Hall R, Mittman BS (2019). Core functions and forms of complex health interventions: a patient-centered medical home illustration. J Gen Intern Med.

[17] **Hawe P, Shiell A, Riley T. In response to Spillane V., Byrne M.C., Byrne M., Leathem C.S., O’Malley M. & Cupples M.E.** (2007) Monitoring treatment fidelity in a randomized trial of a complex intervention. J Adv Nurs 60(3), 343-352. Important considerations for standardizing complex interventions. J Adv Nurs. 2008;62(2):267. 10.1111/j.1365-2648.2008.04686.x 10.1111/j.1365-2648.2008.04686.x 18394039

[18] Villeval M, Bidault E, Shoveller J, Alias F, Basson JC, Frasse C (2016). Enabling the transferability of complex interventions: exploring the combination of an intervention’s key functions and implementation. Int J Public Health.

[19] Villeval M, Gaborit E, Berault F, Lang T, Kelly-Irving M (2019). Do the key functions of an intervention designed from the same specifications vary according to context? Investigating the transferability of a public health intervention in France. Implement Sci IS.

[20] **McGuire AB, Powell KG, Treitler PC, Wagner KD, Smith KP, Cooperman N, et al.** Emergency department-based peer support for opioid use disorder: emergent functions and forms. J Subst Abus Treat 2019. 10.1016/j.jsat.2019.06.013 10.1016/j.jsat.2019.06.013 PMC7393771 31280928

[21] Byng R, Norman I, Redfern S, Jones R (2008). Exposing the key functions of a complex intervention for shared care in mental health: case study of a process evaluation. BMC Health Serv Res.

[22] **Hill J, Cuthel AM, Lin P, Grudzen CR.** Primary Palliative Care for Emergency Medicine (PRIM-ER): applying form and function to a theory-based complex intervention. Contemp Clin Trials Commun 2020:100570. 10.1016/j.conctc.2020.100570 PMC7225617 32426550

[23] Moore GF, Audrey S, Barker M, Bond L, Bonell C, Hardeman W (2015). Process evaluation of complex interventions: Medical Research Council guidance. BMJ (Clin Res Ed).

[24] Butler M, Epstein RA, Totten A, Whitlock EP, Ansari MT, Damschroder LJ (2017). AHRQ series on complex intervention systematic reviews-paper 3: adapting frameworks to develop protocols. J Clin Epidemiol.

[25] Damschroder LJ, Aron DC, Keith RE, Kirsh SR, Alexander JA, Lowery JC (2009). Fostering implementation of health services research findings into practice: a consolidated framework for advancing implementation science. Implement Sci IS.

[26] **Newhouse RP, Himmelfarb CD, Morlock L, Frick KD, Pronovost P, Liang Y.** A phased cluster-randomized trial of rural hospitals testing a quality collaborative to improve heart failure care: organizational context matters. Med Care 2013:396-403. 10.1097/MLR.0b013e318286e32e 23579349

[27] Greenhalgh T, Robert G, Macfarlane F, Bate P, Kyriakidou O (2004). Diffusion of innovations in service organizations: systematic review and recommendations. Milbank Q.

[28] Stirman SW, Baumann AA, Miller CJ (2019). The FRAME: an expanded framework for reporting adaptations and modifications to evidence-based interventions. Implement Sci.

[29] Bauman LJ, Stein RE, Ireys HT (1991). Reinventing fidelity: the transfer of social technology among settings. Am J Community Psychol.

[30] Reelick MF, Faes MC, Esselink RA, Kessels RP, Olde Rikkert MG (2011). How to perform a preplanned process evaluation for complex interventions in geriatric medicine: exemplified with the process evaluation of a complex falls-prevention program for community-dwelling frail older fallers. J Am Med Dir Assoc.

[31] **Craig P, Di Ruggiero E, Frolich KL, Mykhalovskiy E, White M, Campbell R, et al.** Taking account of context in population health intervention research: guidance for producers, users and funders of research. 2018.

[32] AcademyHealth. Evaluating Complex Health Interventions: A Guide to Rigorous Research Designs. 2017.

[33] Minary L, Trompette J, Kivits J, Cambon L, Tarquinio C, Alla F (2019). Which design to evaluate complex interventions? Toward a methodological framework through a systematic review. BMC Med Res Methodol.

[34] Emsley R, Dunn G, White IR (2010). Mediation and moderation of treatment effects in randomised controlled trials of complex interventions. Stat Methods Med Res.

[35] O’Cathain A, Thomas K, Drabble S, Rudolph A, Hewison J (2013). What can qualitative research do for randomised controlled trials? A systematic mapping review. BMJ Open.

[36] Lewin S, Glenton C, Oxman AD (2009). Use of qualitative methods alongside randomised controlled trials of complex healthcare interventions: methodological study. BMJ (Clin Res Ed.).

[37] **Craig P, Matthews L, Moore L, Simpson S, Skivington K.** Updated Guidance: Developing and Evaluating Complex Interventions [Draft for Consultation]. 2019.

