# Supplementary material for: Improving Comparative Effectiveness Research of Complex Health Interventions: Standards from the Patient-Centered Outcomes Research Institute (PCORI)
Source: J Gen Intern Med. 2020 Oct 26;35(Suppl 2):875–81. doi: 10.1007/s11606-020-06093-6 (PMC7652976; doi:10.1007/s11606-020-06093-6)
Supplement: Supplementary file 1 — (DOCX 28 kb) [file 11606_2020_6093_MOESM1_ESM.docx]

*Appendix 1: Literature Review*

*Search Strategy*

We performed an initial PubMed search to identify seminal articles, such as guidelines, frameworks and reviews that address the design and methods of studies that evaluate complex health interventions. The PubMed search covered articles published from 01-01-2005 until 12-01-2016 in the English language:

**Database:** PubMed

**Search strategy:**

("complex interventions"[ti] OR "complex intervention"[ti] OR "multicomponent intervention"[ti] OR

"multicomponent interventions"[ti]) AND ("evaluation"[tw] OR "evaluated"[tw] OR "evaluate"[tw] OR

"evaluating"[tw]) AND (("2005/01/01"[PDAT] : "3000/12/31"[PDAT]) AND English[lang])

**Total results: 215**

Articles that proposed or discussed a guideline, standard, framework or set of principles for evaluating complex interventions or articles that described or discussed the design of, methods for or reporting of evaluations of complex health interventions were included. Specific studies of complex interventions or original research articles were excluded unless they issued guidance or critiqued existing guidance or methodologies.

*Review and selection of relevant literature*

The PCORI staff working group (RB, SA, LE, DH) independently reviewed titles and abstracts and applied the inclusion and exclusion criteria. The group met internally to discuss and resolve the inclusion or exclusion of articles when a second opinion was needed. This yielded 88 references for full text review, which were divided among pairs for full text review. Each pair separately reviewed each full text article, again applying the inclusion and exclusion criteria, meeting to compare results and resolve discrepancies. This step excluded many case studies and original research studies. These steps yielded a total of 44 articles, which are listed below.

From this initial set of documents, four sets of guidelines were selected for formal abstraction.^4,5,12,13^ Two reviewers were assigned to each article to abstract guidance and/or recommendations under each domain. One primary reviewer abstracted the article and the secondary reviewer reviewed the abstractions and refined or revised when needed. The pairs met to discuss and finalize the abstractions. The remaining articles were reviewed but not formally abstracted. These abstractions and background information informed the development of the *initial* set of standards.

Throughout the 18-month standards development process, we added reports, guidance documents and articles as recommended by other PCORI staff, PCORI Methodology Committee members, external experts, and through the review of reference lists. This process yielded an additional 21 references. We consulted a total of 65 articles.

*Updated Literature Review: Bridge Search*

Given the rapid developments in this field, we performed an updated bridge search of PubMed in August 2019. This search, which is outlined below, revealed an additional 107 articles. None of these included new guidance; but some outlined an initiative to update and develop new guidance.^69^ The most relevant articles from this search are listed below.

**Updated bridge search (date run: 8/21/19)**

**Database:** PubMed

**Search strategy:**

("complex interventions"[ti] OR "complex intervention"[ti] OR "multicomponent intervention"[ti] OR

"multicomponent interventions"[ti]) AND ("evaluation"[tw] OR "evaluated"[tw] OR "evaluate"[tw] OR

"evaluating"[tw]) AND (("2005/01/01"[PDAT] : "3000/12/31"[PDAT]) AND English[lang])

**Total Results: 324**

**Total results since 12/1/16:** 107

**List of Articles Consulted to Develop the Standards for Studies of Complex Interventions**

**Guidance Documents**

1. Butler M, Epstein RA, Totten A, Whitlock EP, Ansari MT, Damschroder LJ, Balk E, Bass EB, Berkman ND, Hempel S, Iyer S. AHRQ series on complex intervention systematic reviews—paper 3: adapting frameworks to develop protocols. *Journal of clinical epidemiology*. 2017 Oct 1;90:19-27.
2. Boutron I, Moher D, Altman DG, Schulz KF, Ravaud P. Extending the CONSORT statement to randomized trials of nonpharmacologic treatment: explanation and elaboration. Annals of internal medicine. 2008 Feb 19;148(4):295-309.
3. Chan AW, Tetzlaff JM, Altman DG, Laupacis A, Gøtzsche PC, Krleža-Jerić K, Hróbjartsson A, Mann H, Dickersin K, Berlin JA, Doré CJ. SPIRIT 2013 statement: defining standard protocol items for clinical trials. Annals of internal medicine. 2013 Feb 5;158(3):200-7.
4. Craig P, Dieppe P, Macintyre S, Michie S, Nazareth I, Petticrew M. Developing and evaluating complex interventions: the new Medical Research Council guidance. *International Journal of Nursing Studies.* 2013 May 31; 50(5):587-92.
5. Craig P, Dieppe P, Macintyre S, Michie S, Nazareth I, Petticrew M. Developing and evaluating complex interventions: the new Medical Research Council guidance. *BMJ*. 2008 Sep 29; 337:a1655.
6. Damschroder LJ, Aron DC, Keith RE, Kirsh SR, Alexander JA, Lowery JC. Fostering implementation of health services research findings into practice: a consolidated framework for advancing implementation science. *Implementation science*. 2009 Dec;4(1):50.
7. Guise JM, Butler ME, Chang C, Viswanathan M, Pigott T, Tugwell P, Workgroup CI. AHRQ series on complex intervention systematic reviews—paper 6: PRISMA-CI extension statement and checklist. *Journal of clinical epidemiology*. 2017 Oct 1;90:43-50.
8. Guise JM, Butler M, Chang C, Viswanathan M, Pigott T, Tugwell P, Workgroup CI. AHRQ series on complex intervention systematic reviews—paper 7: PRISMA-CI elaboration and explanation. *Journal of clinical epidemiology*. 2017 Oct 1;90:51-8.
9. Guise JM, Chang C, Butler M, Viswanathan M, Tugwell P. AHRQ series on complex intervention systematic reviews—paper 1: an introduction to a series of articles that provide guidance and tools for reviews of complex interventions. *Journal of clinical epidemiology*. 2017 Oct 1;90:6-10.
10. Kelly MP, Noyes J, Kane RL, Chang C, Uhl S, Robinson KA, Springs S, Butler ME, Guise JM. AHRQ series on complex intervention systematic reviews—paper 2: defining complexity, formulating scope, and questions. *Journal of clinical epidemiology*. 2017 Oct 1;90:11-8.
11. Möhler R, Bartoszek G, Köpke S, Meyer G. Proposed criteria for reporting the development and evaluation of complex interventions in healthcare (CReDECI): guideline development. *International Journal of Nursing Studies.* 2012 Jan 31; 49(1):40-6.
12. Möhler R, Köpke S, Meyer G. Criteria for Reporting the Development and Evaluation of Complex Interventions in healthcare: revised guideline (CReDECI 2*). Trials.* 2015 May 3; 16(1):204.
13. Moore GF, Audrey S, Barker M, Bond L, Bonell C, Hardeman W, Moore L, O’Cathain A, Tinati T, Wight D, Baird J. Process evaluation of complex interventions: Medical Research Council guidance. *BMJ.* 2015 Mar 19; 350:h1258.
14. Moore G, Audrey S, Barker M, Bond L, Bonell C, Hardeman W, Moore L, O’Cathain A, Tinati T, Wight D, Baird J. Process evaluation of complex interventions: Medical Research Council guidance. MRC Population Health Science Research Network. 2014.
15. Ogrinc G, Davies L, Goodman D, Batalden P, Davidoff F, Stevens D. SQUIRE 2.0 (Standards for QUality Improvement Reporting Excellence): revised publication guidelines from a detailed consensus process. *The Journal of Continuing Education in Nursing*. 2015 Oct 26;46(11):501-7.
16. Pigott, T., Noyes, J., Umscheid, C.A., Myers, E., Morton, S.C., Fu, R., Sanders-Schmidler, G.D., Devine, B., Murad, M.H., Kelly, M.P. and Fonnesbeck, C., 2017. AHRQ series on complex intervention systematic reviews—paper 5: advanced analytic methods. *Journal of clinical epidemiology*, 90, pp.37-42.
17. Pinnock H, Barwick M, Carpenter CR, Eldridge S, Grandes G, Griffiths CJ, Rycroft-Malone J, Meissner P, Murray E, Patel A, Sheikh A. Standards for reporting implementation studies (StaRI) statement. *BMJ*. 2017 Mar 6;356:i6795.
18. Stirman SW, Miller CJ, Toder K, Calloway A. Development of a framework and coding system for modifications and adaptations of evidence-based interventions. *Implementation Science*. 2013 Dec;8(1):65.
19. Viswanathan M, McPheeters ML, Murad MH, Butler ME, Devine EE, Dyson MP, Guise JM, Kahwati LC, Miles JN, Morton SC. AHRQ series on complex intervention systematic reviews—paper 4: selecting analytic approaches. *Journal of clinical epidemiology*. 2017 Oct 1;90:28-36.

**Background Documents**

1. Anderson R. New MRC guidance on evaluating complex interventions. *BMJ.* 2008 Oct 22; 337:a1937.
2. Bauman, L.J., Stein, R.E. and Ireys, H.T., 1991. Reinventing fidelity: The transfer of social technology among settings. American Journal of Community Psychology, 19(4), pp.619-639.
3. Blackwood B. Methodological issues in evaluating complex healthcare interventions. *Journal of Advanced Nursing.* 2006 Jun 1; 54(5):612-22.
4. Bonell, C., Fletcher, A., Morton, M., Lorenc, T. and Moore, L., 2012. Realist randomised controlled trials: a new approach to evaluating complex public health interventions. *Social science & medicine*, *75*(12), pp.2299-2306.
5. Bonetti D, Clarkson JE. The challenges of designing and evaluating complex interventions. *Community Dental Health*. 2010 Sep; 27(3):130.
6. Byng, R., Norman, I., Redfern, S. and Jones, R., 2008. Exposing the key functions of a complex intervention for shared care in mental health: case study of a process evaluation. BMC Health Services Research, 8(1), p.274.
7. Campbell NC, Murray E, Darbyshire J, Emery J, Farmer A, Griffiths F, Guthrie B, Lester H, Wilson P, Kinmonth AL. Designing and evaluating complex interventions to improve health care. *BMJ*. 2007 Mar 3; 334:455-9.
8. Chen YF, Hemming K, Stevens AJ, Lilford RJ. Secular trends and evaluation of complex interventions: the rising tide phenomenon. *BMJ Quality & Safety.* 2016 May; 25(5):303-10.
9. Coly, A., & Parry, G. “Evaluating Complex Health Interventions: A Guide to Rigorous Research Designs,” AcademyHealth, June 2017.
10. Craig P, Petticrew M. Developing and evaluating complex interventions: reflections on the 2008 MRC guidance. *International Journal of Nursing Studies.* 2013 May 31; 50(5):585-7.
11. Datta J, Petticrew M. Challenges to evaluating complex interventions: a content analysis of published papers. *BMC Public Health.* 2013 Jun 11; 13(1):568.
12. De Silva MJ, Breuer E, Lee L, Asher L, Chowdhary N, Lund C, Patel V. Theory of Change: a theory-driven approach to enhance the Medical Research Council's framework for complex interventions. *Trials.* 2014 Jul 5; 15(1):267.
13. Dudley, RA, Struhl, B. Evaluating Complex Health Services Interventions: Challenges, Goals, and Proposals for Progress. *AcademyHealth.* 2016 Jun.
14. Dunn G, Emsley R, Liu H, Landau S, Green J, White I, et al. Evaluation and validation of social and psychological markers in randomised trials of complex interventions in mental health: a methodological research programme. Health Technol Assess 2015;19(93).
15. Emsley R, Dunn G, White IR. Mediation and moderation of treatment effects in randomised controlled trials of complex interventions. *Statistical Methods in Medical Research.* 2010 Jun 1; 19(3):237-70.
16. Fletcher A, Jamal F, Moore G, Evans RE, Murphy S, Bonell C. Realist complex intervention science: Applying realist principles across all phases of the Medical Research Council framework for developing and evaluating complex interventions*. Evaluation*. 2016 Jul; 22(3):286-303.
17. Guise JM, Chang C, Viswanathan M, Glick S, Treadwell J, Umscheid CA, Whitlock E, Fu R, Berliner E, Paynter R, Anderson J. Systematic reviews of complex multicomponent health care interventions. Research White Paper. AHRQ Publication No. 14-EHC003- EF. Rockville, MD: Agency for Healthcare Research and Quality. March 2014.
18. Grant A, Treweek S, Dreischulte T, Foy R, Guthrie B. Process evaluations for cluster-randomised trials of complex interventions: a proposed framework for design and reporting. *Trials.* 2013 Jan 12; 14(1):15.
19. Greenhalgh, T., Robert, G., Macfarlane, F., Bate, P. and Kyriakidou, O., 2004. Diffusion of innovations in service organizations: systematic review and recommendations. The Milbank Quarterly, 82(4), pp.581-629.
20. Harris FM, Maxwell M, O’Connor R, Coyne JC, Arensman E, Coffey C, Koburger N, Gusmão R, Costa S, Székely A, Cserhati Z. Exploring synergistic interactions and catalysts in complex interventions: longitudinal, mixed methods case studies of an optimised multi-level suicide prevention intervention in four european countries (Ospi-Europe). *BMC Public Health.* 2016 Mar 15; 16(1):268.
21. Hasson H. Systematic evaluation of implementation fidelity of complex interventions in health and social care. *Implementation Science.* 2010 Sep 3; 5(1):67.
22. Hawe P. Lessons from complex interventions to improve health. *Annual Review of Public Health.* 2015 Mar 18; 36:307-23.
23. Hawe P, Shiell A, Riley T. Complex interventions: how" out of control" can a randomised controlled trial be? *BMJ.*  2004 Jun 26; 328(7455):1561.
24. Hawe P, Shiell A, Riley T. In response to Spillane V., Byrne MC, Byrne M., Leathem CS, O’Malley M. & Cupples ME (2007) Monitoring treatment fidelity in a randomized trial of a complex intervention. Journal of Advanced Nursing60 (3), 343–352. *Journal of Advanced Nursing*. 2008 Apr;62(2):267-
25. Haynes A, Brennan S, Carter S, O'Connor D, Schneider CH, Turner T, Gallego G. Protocol for the process evaluation of a complex intervention designed to increase the use of research in health policy and program organisations (the SPIRIT study). *Implementation Science.* 2014 Sep 27; 9(1):113.
26. Higginson IJ, Evans CJ, Grande G, Preston N, Morgan M, McCrone P, Lewis P, Fayers P, Harding R, Hotopf M, Murray SA. Evaluating complex interventions in end of life care: the MORECare statement on good practice generated by a synthesis of transparent expert consultations and systematic reviews. *BMC medicine.* 2013 Apr 24; 11(1):111.
27. Hoffmann TC, Glasziou PP, Boutron I, Milne R, Perera R, Moher D, Altman DG, Barbour V, Macdonald H, Johnston M, Lamb SE. Better reporting of interventions: template for intervention description and replication (TIDieR) checklist and guide. *BMJ.* 2014 Mar 7; 348:g1687.
28. Kane, H., Lewis, M.A., Williams, P.A. and Kahwati, L.C., 2014. Using qualitative comparative analysis to understand and quantify translation and implementation. *Translational behavioral medicine*, 4(2), pp.201-208.
29. Komro KA, Flay BR, Biglan A, Wagenaar AC. Research design issues for evaluating complex multicomponent interventions in neighborhoods and communities. *Translational Behavioral Medicine.* 2016 Mar 1; 6(1):153-9.
30. Lancaster GA, Campbell MJ, Eldridge S, Farrin A, Marchant M, Muller S, Perera R, Peters TJ, Prevost AT, Rait G. Trials in primary care: statistical issues in the design, conduct and evaluation of complex interventions. *Statistical Methods in Medical Research.* 2010 Aug; 19(4):349-77.
31. Liu H, Muhunthan J, Hayek A, Hackett M, Laba TL, Peiris D, Jan S. Examining the use of process evaluations of randomised controlled trials of complex interventions addressing chronic disease in primary health care—a systematic review protocol. *Systematic Reviews.* 2016 Aug 15; 5(1):138.
32. May C, Finch T, Mair F, Ballini L, Dowrick C, Eccles M, Gask L, MacFarlane A, Murray E, Rapley T, Rogers A. Understanding the implementation of complex interventions in health care: the normalization process model. *BMC Health Services Research.* 2007 Sep 19; 7(1):148.
33. May CR, Mair FS, Dowrick CF, Finch TL. Process evaluation for complex interventions in primary care: understanding trials using the normalization process model. *BMC Family Practice.* 2007 Jul 24; 8(1):42.
34. Movsisyan A, Melendez-Torres GJ, Montgomery P. Outcomes in systematic reviews of complex interventions never reached “high” GRADE ratings when compared with those of simple interventions. *Journal of Clinical Epidemiology.* 2016 Oct 31; 78:22-33.
35. Murray E, Treweek S, Pope C, MacFarlane A, Ballini L, Dowrick C, Finch T, Kennedy A, Mair F, O'Donnell C, Ong BN. Normalisation process theory: a framework for developing, evaluating and implementing complex interventions. *BMC Medicine*. 2010 Oct 20; 8(1):63.
36. Noyes J, Gough D, Lewin S, Mayhew A, Michie S, Pantoja T, Petticrew M, Pottie K, Rehfuess E, Shemilt I, Shepperd S. A research and development agenda for systematic reviews that ask complex questions about complex interventions. *Journal of Clinical Epidemiology.* 2013 Nov 30; 66(11):1262-70.
37. Petticrew M, Anderson L, Elder R, Grimshaw J, Hopkins D, Hahn R, Krause L, Kristjansson E, Mercer S, Sipe T, Tugwell P. Complex interventions and their implications for systematic reviews: a pragmatic approach. *International Journal of Nursing Studies.* 2015 Jul; 52(7):1211-6.
38. Petticrew M, Anderson L, Elder R, Grimshaw J, Hopkins D, Hahn R, Krause L, Kristjansson E, Mercer S, Sipe T, Tugwell P. Complex interventions and their implications for systematic reviews: a pragmatic approach. *Journal of Clinical Epidemiology.* 2013 Nov 30; 66(11):1209-14.
39. Pinnock H, Epiphaniou E, Taylor SJ. Phase IV implementation studies. The forgotten finale to the complex intervention methodology framework. *Annals of the American Thoracic Society*. 2014 Feb; 11(Supplement 2):S118-22.
40. Raine R, Fitzpatrick R, Barratt HS, Campbell M, Moore L, Zwarenstein M, Bower P. Challenges, solutions and future directions in the evaluation of service innovations in health care and public health. *Health Services and Delivery Research.* 2016; 4(16).
41. Reynolds J, DiLiberto D, Mangham-Jefferies L, Ansah EK, Lal S, Mbakilwa H, Bruxvoort K, Webster J, Vestergaard LS, Yeung S, Leslie T. The practice of ‘doing’evaluation: lessons learned from nine complex intervention trials in action. *Implementation Science.* 2014 Jun 17; 9(1):75.
42. Richards DA, Hallberg IR. Complex interventions in health: an overview of research methods. *Routledge*; 2015 Apr 17.
43. Rickles D. Causality in complex interventions. *Medicine, Health Care and Philosophy*. 2009 Feb 1; 12(1):77-90.
44. Shepperd S, Lewin S, Straus S, Clarke M, Eccles MP, Fitzpatrick R, Wong G, Sheikh A. Can we systematically review studies that evaluate complex interventions? *PLoS Med.* 2009 Aug 11; 6(8):e1000086.
45. Shiell A, Hawe P, Gold L. Complex interventions or complex systems? Implications for health economic evaluation. *BMJ.* 2008 Jun 7:1281-3.
46. Wells M, Williams B, Treweek S, Coyle J, Taylor J. Intervention description is not enough: evidence from an in-depth multiple case study on the untold role and impact of context in randomised controlled trials of seven complex interventions. *Trials.* 2012 Jun 28; 13(1):95.

Relevant Articles From Bridge Search (August 2019)

1. Alan B. McGuire, et al. Emergency department-based peer support for opioid use disorder: Emergent functions and forms. Journal of Substance Abuse Treatment. <https://doi.org/10.1016/j.jsat.2019.06.013>
2. Bleijenberg N, de Man-van Ginkel JM, Trappenburg JCA, Ettema RGA, Sino CG, Heim N, et al. Increasing value and reducing waste by optimizing the development of complex interventions: Enriching the development phase of the Medical Research Council (MRC) Framework. International journal of nursing studies. 2018;79:86-93. doi:10.1016/j.ijnurstu.2017.12.001
3. Minary L, Trompette J, Kivits J, Cambon L, Tarquinio C, Alla F. Which design to evaluate complex interventions? Toward a methodological framework through a systematic review. BMC medical research methodology. 2019;19(1):92. doi:10.1186/s12874-019-0736-6
4. Skivington K, Matthews L, Craig P, Simpson S, Moore L. Developing and evaluating complex interventions: updating Medical Research Council guidance to take account of new methodological and theoretical approaches. The Lancet. 2018;392:S2. doi:10.1016/S0140-6736(18)32865-4
5. Villeval M, Bidault E, Shoveller J, Alias F, Basson JC, Frasse C, et al. Enabling the transferability of complex interventions: exploring the combination of an intervention's key functions and implementation. International journal of public health. 2016;61(9):1031-8. doi:10.1007/s00038-016-0809-9
