# Supplementary material for: Improving Comparative Effectiveness Research of Complex Health Interventions: Standards from the Patient-Centered Outcomes Research Institute (PCORI)
Source: J Gen Intern Med. 2020 Oct 26;35(Suppl 2):875–81. doi: 10.1007/s11606-020-06093-6 (PMC7652976; doi:10.1007/s11606-020-06093-6)
Supplement: Supplementary file 2 — (DOCX 19 kb) [file 11606_2020_6093_MOESM2_ESM.docx]

**Appendix 2: Final Methodology Standards for Studies of Complex Interventions^[[1]](#footnote-1)^**

**SCI-1: Fully describe the intervention and comparator and define their core functions.**
Describe the intervention and comparator under study and clearly define aspects related to core functions and forms. Core functions refer to the intended purpose(s) of the interventions. The form of the interventions includes the intended modes of delivery, providers involved, materials or tools required, dose, and frequency/intensity. The description should also explicitly indicate to whom the intervention is aimed (e.g., patient, provider, hospital, health system).

**SCI-2: Specify the hypothesized causal pathways and their theoretical basis.**
Clearly describe the hypothesized causal pathways by which the proposed complex intervention generates change (**see CI-1**). This description should depict how each intervention function generates the hypothesized effects on the prespecified patient outcome(s). Include in the causal model key contextual factors that may influence the impact of the intervention so that their hypothesized relationships are made explicit. Describe the theoretical and/or empirical bases underlying the proposed interventions and their hypothesized effects.

**SCI-3: Specify how adaptations to the form of the intervention and comparator will be allowed and recorded.**
Specify any allowable adaptations in form and describe how planned and unplanned adaptations will be managed, measured/documented, and reported over time. Any planned adaptations should have a clear rationale; be supported by theory, evidence, or experience; and maintain fidelity to the core functions of the intervention. Upon conclusion of the study, researchers should provide guidance on allowable adaptations or unproductive adaptations (i.e., adaptations that may reduce the effectiveness of an intervention).

**SCI-4: Plan and describe a process evaluation.**
Describe plans to conduct a process evaluation (i.e., to assess whether the intervention was implemented as planned and to test and refine the hypothesized causal pathways). Process evaluations should measure/document, analyze, and report the fidelity of the delivery of the intervention (i.e., planned and unplanned adaptations); the quantity or dose of the intervention actually delivered; whether the intended population(s) received the delivered intervention (i.e., reach); the mechanisms of action (e.g., mediators, intermediate outcomes); and important contextual factors (e.g., moderators), taking into account the levels at which the intervention is aimed (e.g., patient, provider, hospital).

Researchers should select a combination of methods appropriate to the process questions identified and describe the timing and sources of data collection. These plans should include appropriate quantitative, qualitative, and/or mixed methods that account for the intervention functions as defined by the causal pathway.

Describe the plans for integration of process and outcome data in advance of intervention delivery to determine whether and how outcomes and effects are influenced by implementation or contextual moderators. Explain how the results of the process evaluation will be used to draw inferences about both effectiveness (i.e., patient outcomes) and the processes of care (i.e., process outcomes).

**SCI-5: Select patient outcomes informed by the causal pathway.**
Select valid and reliable patient outcome measures that are explicitly affected by the hypothesized causal pathway and the theoretical and/or empirical basis for the intervention. If the study does not measure a patient outcome, researchers must provide strong evidence supporting the linkage between the measured outcome and unmeasured patient outcome. The outcome measures should assess the intervention across a range of domains that sufficiently permit assessment of how the intervention affects patients. In determining the length of follow-up, assumptions about the rate and pattern of change expected in the outcome measures should be clear.

1. Available at: https://www.pcori.org/research-results/about-our-research/research-methodology/pcori-methodology-standards#Complex [↑](#footnote-ref-1)
